# Downregulated Expression of linc-ROR in Gastric Cancer and Its Potential Diagnostic and Prognosis Value

**DOI:** 10.1155/2020/7347298

**Published:** 2020-10-24

**Authors:** Xiuchong Yu, Haixiang Ding, Yijiu Shi, Liangwei Yang, Jiaming Zhou, Zhilong Yan, Bingxiu Xiao

**Affiliations:** ^1^Department of Gastrointestinal Surgery, Ningbo First Hospital, Ningbo 315010, China; ^2^Department of Biochemistry and Molecular Biology and Zhejiang Key Laboratory of Pathophysiology, Medical School of Ningbo University, Ningbo 315211, China

## Abstract

**Background:**

Gastric cancer (GC) is one of the global mortality diseases and has a poor prognosis due to the lack of ideal tumor biomarkers. Numerous studies have shown that long noncoding RNAs (lncRNAs) can affect the occurrence and development of cancer through a variety of signaling pathways. The abnormal expression and specificity of lncRNAs in tumors make them potential biomarkers of cancers. Nevertheless, the diagnostic roles of lncRNAs in GC have been poorly understood. So this study focuses on the clinical diagnostic value of lncRNAs in GC.

**Materials and Methods:**

Quantitative reverse transcription-polymerase chain reaction (qRT-PCR) was used to investigate the expression of the linc-ROR (long intergenic noncoding RNA, regulator of reprogramming) in 105 paired GC tissues and adjacent normal tissues. Receiver operating characteristic (ROC) curve and area under the curve (AUC) were established to assess the diagnostic value of linc-ROR. The relationship between expression of linc-ROR and clinicopathological factors of patients with GC was further explored. Kaplan-Meier analysis was performed to evaluate the prognostic value of linc-ROR expression.

**Results:**

The linc-ROR expression level was significantly decreased in GC tissues compared with its adjacent nontumor tissues (*n* = 105, *P* < 0.001). We also discovered that linc-ROR was evidently downregulated in 68.6% (72/105) of GC tissues. The AUC's value of linc-ROR was up to 0.6495, with sensitivity and specificity of 0.7524 and 0.5143, respectively. Intriguingly, the linc-ROR expression levels were obviously associated with tumor differentiation (*P* = 0.004). Notably, the overall survival rate of GC patients with high expression of linc-ROR was significantly higher than those with low expression.

**Conclusion:**

Our data revealed that linc-ROR has clinical potential as a biomarker for the diagnosis of GC and assessment of its prognosis.

## 1. Introduction

GC is a very common malignant tumor with a high mortality rate, especially in Southeast Asian countries^1^–^3^. With the development of modern medicine, the overall survival rate of patients with GC has improved, but most patients with early-stage gastric cancer (EGC) have no obvious characteristic performance^4^, ^5^. Unfortunately, most patients are diagnosed at an advanced stage and missed the best treatment time. In the diagnosis of GC, upper gastrointestinal (GI) endoscopy is the gold standard for the diagnosis of GC^6^, but endoscopy is an invasive technique that can cause pain to the patient and this is not a routine examination^7^. Importantly, the 5-year survival rate of patients with EGC can reach more than 90% and the survival rate of patients with metastatic gastric cancer is just only 18%^8^, ^9^. Clinically, the sensitivity and specificity of traditional tumor markers such as carcinoembryonic antigen (CEA), carbohydrate antigen 19-9 (CA19-9), and CA72-4 are relatively low^10^–^12^. Therefore, it is particularly important and urgent to find minimally invasive or even noninvasive biomarkers that can diagnose GC early.

More than 70% of the human genome can be transcribed, and most of them are transcribed into noncoding RNAs, including circular RNAs (circRNAs), long noncoding RNAs (lncRNAs), microRNAs (miRNAs), and so on^13^–^15^. Among them, lncRNAs are a type of noncoding RNA of more than 200 nt in length. Because they do not have the ability to encode proteins, they were once considered to be the noise of gene transcription^16^. However, studies have shown that lncRNAs, as a regulatory molecule in gene expression, are directly involved in the development of various human diseases, especially various cancers^17^–^19^. lncRNAs can be used as oncogenes or tumor suppressor genes to participate in the growth, proliferation, metastasis, and drug resistance of GC^20^. In addition, long intergenic (or intermediate) ncRNAs (lincRNAs) are located between the coding and noncoding genes and do not overlap with the exons of other genes. Besides, the characteristic SNP (single nucleotide polymorphism, SNP) content of lincRNAs is high, indicating that they can play a role in the characteristic correlation regions between genes^21^, ^22^. Because of their unique secondary structure and their high stability and specificity, lncRNAs can be stably present in body fluids (such as blood, saliva, and urine). Therefore, free lncRNAs in body fluids can be determined by quantitative detection methods. In addition, more and more researches show that the abnormal expression of lncRNA has clinical significance for the diagnosis of GC^23^–^25^. Zhang et al. identified five lincRNAs from the plasma of GC patients, which can be distinguished between GC patients and healthy people via using five lncRNAs as joint indicators^26^. Another study also found that lncRNA ZNFX1-AS1 and HULC are differentially expressed in the plasma of GC patients and healthy people, indicating that ZNFX1-AS1 and HULC are promising in the clinical diagnosis of GC^27^. lincRNA reprogramming regulator (linc-ROR) can regulate iPSC reprogramming^28^. The study found that linc-ROR can respond to DNA damage by interacting with heterogeneous ribonucleoprotein I (hnRNP I) to inhibit p53 translation^29^. Besides, linc-ROR promotes the growth of breast cancer cells independent of estrogen^30^. Importantly, linc-ROR has a potential role in the diagnosis of cancer. Upregulated linc-ROR may be a potential biomarker for the diagnosis and dynamic monitoring of breast cancer^31^. The existing researches about linc-ROR indicate that it not only plays an important role in the occurrence and development of tumors but also has considerable potential in tumor diagnosis.

In this study, our purpose was to find early tumor biomarkers that can distinguish GC patients from healthy people. qRT-PCR was used to investigate the expression of linc-ROR in 105 pairs of GC tissues and its corresponding adjacent tissues. Additionally, we have constructed a receiver operating characteristic (ROC) curve and survival curves to assess its potential for diagnosis and prognosis of GC markers. The results revealed that the expression of linc-ROR in GC tissues was significantly lower than that of adjacent normal tissues. The area under curve (AUC) also disclosed that linc-ROR has potential as a diagnostic marker, and the Kaplan-Meier plotter showed that the survival rate of GC patients with high expression of linc-ROR was significantly higher than that of patients with low expression of linc-ROR.

## 2. Materials and Methods

### 2.1. Specimens and Clinical Data

The 105 GC tissues and paired adjacent nontumorous tissues were collected from the Yinzhou People's Hospital in Ningbo between January 2010 and December 2015. All tissue specimens were obtained from GC patients who had not undergone any treatment before surgery, and the GC tissues were taken from the mucosa of the tumor center, more than 5 cm from the adjacent tissues. Specimens were removed from the patient with GC, then placed in a solution (Bioteke, China) at once to avoid RNA degradation. Finally, they were stored at -80°C until use. All specimens were eventually confirmed as GC according to histopathology, and the tumor staging was determined by the International Union Against Cancer's Tumor-Node-Metastasis (TNM) staging system version 8^32^, ^33^. Assessment of histological grades was according to the National Comprehensive Cancer Network (NCCN) Cancer Clinical Practice Guidelines (V.1.2011)^34^. Written informed consent was obtained from all patients, and the study protocol was approved by the Human Research Ethics Committee of Ningbo First Hospital (IRB No. 2019-R012). All experiments follow the appropriate guidelines and regulations.

### 2.2. Total RNA Extraction

Total RNA of GC tissue sample extraction was performed using a TRIzol reagent (Invitrogen, Karlsruhe, Germany) following the manufacturer's protocol. Subsequently, the quality and concentration of RNA were measured by a NanoDrop spectrophotometer (DeNovix, Wilmington, USA). The absorbance ratio of RNA A260/A280 should be between 1.8 and 2.0 for subsequent experiments. The RNA with the desired purity was reverse transcribed into complementary DNA (cDNA), and the remaining was stored in -80°C for backup.

### 2.3. Reverse Transcription

2 *μ*g of total RNA was performed to synthesize cDNA using the GoScript RT System (Promega, Madison, WI, USA) with random primers according to the manufacturer's instructions.

### 2.4. Real-Time Polymerase Chain Reaction

We used the GoTaq qPCR Master (Promega, USA) for qRT-PCR. The reaction volume was 25 *μ*l, including 12.5 *μ*l of GoTaq® qPCR Master Mix (2x), 1 *μ*l of forward primer (10 *μ*M), 1 *μ*l of reverse primer (10 *μ*M), 5 *μ*l of cDNA, and 5.5 *μ*l of RNase-free ddH_2_O on the Mx3005P real-time PCR system (Stratagene, USA). The sequences of qRT-PCR for linc-ROR were 5′-CTTGATGGCATTGTCGC-3′ and 5′-TCCTGTGGTTTCATTGTCC-3′. And *β*-actin was performed as the internal negative control and normalized the levels of linc-ROR. The sequences of *β*-actin were 5′-AAGCCACCCCACTTCTCTCTAA-3′ and 5′-AATGCTATCACCTCCCCTGTGT-3′. The reaction mixtures were incubated at 95°C for 5 min, followed by 40 cycles at 95°C for 15 s, 58°C for 30 s, and 72°C for 30 s. The melt curve was used to verify the specificity of the qRT-PCR products. All experiments were carried out in triplicate. Each sample amplified specific linc-ROR and *β*-actin RNA. The ΔC*q* method was used for analysis. First, we detect the C*q* value of each sample through the Mx3005P QPCR System. Then, we calculated ΔC*q* values according to the formula ΔC*q* = C*q*_linc‐ROR_ − C*q*_*β*‐actin_; ΔΔC*q* value is determined by the formula ΔΔC*q* = ΔC*q*_gastric cancer group_ − ΔC*q*_adjacent normal group_. The relative expression level of linc-ROR in adjacent normal tissues and gastric cancer tissues was determined using the ΔC*q* method^34^. If the ΔC*q* value is relatively high, the expression of linc-ROR is relatively low^35^. The log2^−ΔΔC*q*^ < 0 was used to analyze the relative lower linc-ROR expression samples in gastric cancer tissues.

### 2.5. Statistical Analysis

Statistical calculations were analyzed by the Statistical Product and Service Solutions 19.0 software (SPSS, Chicago, IL, USA). For comparing the expression of linc-ROR between GC tissues and paired adjacent nontumor tissues, two-tailed Student's *t*-test was used. ROC and AUC were used to evaluate the diagnostic value of linc-ROR. A chi-square test was used to compare the relationship between the expression of linc-ROR and pathological factors in patients with GC. Results were considered statistically significant when the *P* value was less than 0.05.

## 3. Results

### 3.1. linc-ROR Was Downregulated in GC Tissues

**Figure 1:**
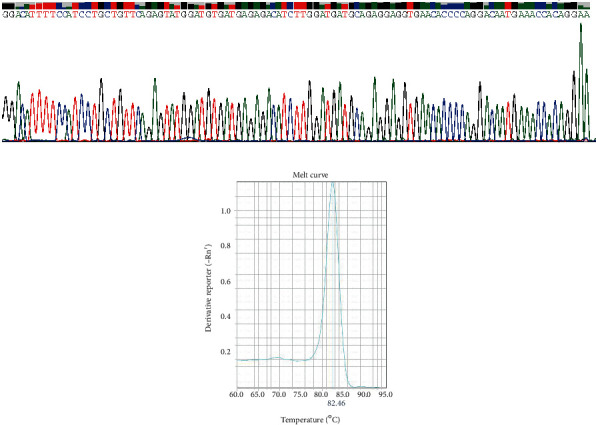
Amplification of linc-ROR. (a) Sanger sequence results of qRT-PCR products of linc-ROR in GC tissues. (b) The single peak of the melting curve of linc-ROR. qRT-PCR: quantitative reverse transcription-polymerase chain reaction.

**Figure 2:**
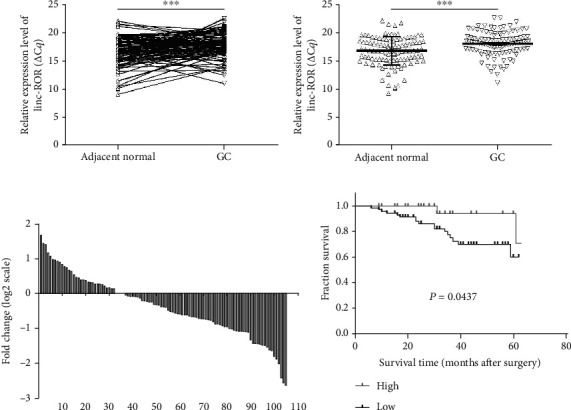
The relative expression level of linc-ROR in gastric cancer tissues. Statistical significance was defined as two-sided. ^∗∗∗^*P* < 0.001. (a) Expression levels of linc-ROR in each patient, with comparison between tumor tissues and the normal adjacent tissues (*n* = 105). Higher ΔC*q* value indicates lower expression. ^∗∗∗^*P* < 0.001. (b) The expression levels of linc-ROR were significantly lower than those in adjacent normal tissues (*n* = 105, *P* < 0.001). (c) The percentage of low expression of linc-ROR in GC tissues accounts for 68.6% (72/105). (d) Kaplan-Meier analysis of OS based on linc-ROR expression in all GC patients.

To determine the expression of linc-ROR in GC tissues, we analyzed their expression in 105 pairs of GC tissues and corresponding adjacent tissues by qRT-PCR. The Sanger sequence results of the qRT-PCR product (165 bp) were consistent with the original sequence (Figure 1(a)), and the single peak of the melting curve of linc-ROR verified the specificity of the primer (Figure 1(b)). As a result, the expression level of linc-ROR in GC tissues was much lower than that in the corresponding adjacent cancer tissues (Figures 2(a) and 2(b)). Among all samples, the lower expression samples accounted for more than 68.6% (72/105, Figure 2(c)). Correlation analysis showed that the expression of linc-ROR in the GC group was related to the degree of differentiation (*P* = 0.004). However, linc-ROR expression level was not correlated with age (*P* = 0.324), gender (*P* = 0.748), tumor size (*P* = 0.767), lymph node metastasis (*P* = 0.807), invasion (*P* = 0.675), distant metastasis (*P* = 0.811), TNM stage (*P* = 0.291), CEA (*P* = 0.392), and CA19-9 (*P* = 0.509) (Table 1).

### 3.2. linc-ROR Has Potential as a Diagnostic and Prognostic Biomarker in GC

**Figure 3:**
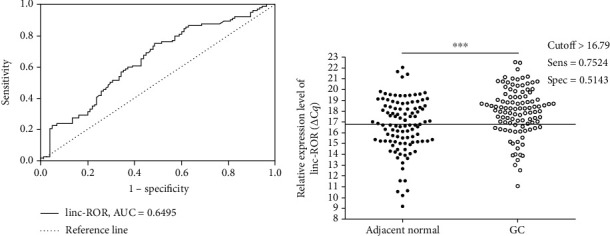
The diagnostic values of linc-ROR in gastric cancer. (a) The ROC curve of linc-ROR in differentiating GC tissues from controls. The area under the curve was up to 0.6495. **(**b**)** The cutoff value, sensitivity, and specificity were established by ROC curve. ROC: receiver operating characteristic.

To investigate the prognosis of linc-ROR in GC, we analyzed the survival curve to show that patients with higher linc-ROR expression had a longer overall survival and vice versa (*P* = 0.0437; Figure 2(d)). We further evaluated the diagnostic value of linc-ROR in GC tissues and corresponding adjacent tissues by constructing ROC curves. Analysis of the ROC curve results showed that the AUC value of linc-ROR was 0.6495 (Figure 3(a)), the cutoff value was greater than 16.79, with sensitivity and specificity 0.7524 and 0.5143, respectively (Figure 3(b)).

### 3.3. linc-ROR Has the Potential to Bind RBP to Exert Biological Functions

A large number of studies have shown that lncRNAs can act as a molecular sponge or bait of RNA binding protein (RBP) through its RBP binding site, thereby regulating the expression of all target genes of the corresponding target RBP^36^, ^37^. We have found two RBPs (ADAR and FUS) that can be combined with linc-ROR (Table 2) through the bioinformatic database starBase v3.0 (http://starbase.sysu.edu.cn/) laying the foundation for future research on whether or not to play a biological role in GC.

## 4. Discussion

Although the current diagnosis and treatment of GC has made a breakthrough, GC is still the third leading cause of cancer death; the patient's 5-year survival rate is also very low^38^. Since reducing mortality is closely related to early diagnosis of gastric cancer, there is an urgent need to identify new tumor biomarkers for early diagnosis so that patients can be treated within the optimal treatment time and improve survival. In the clinical diagnosis of GC, gastroscopy is the gold standard, but it is invasive and the patient feels uncomfortable. Additionally, the effect of traditional tumor biomarkers is not ideal. So, minimally invasive liquid biopsy has become the mainstream of finding tumor markers, and lncRNAs have a potential role in the diagnosis of cancer^39^. We found that the expression levels of linc-ROR were lower in GC tissues compared with its adjacent normal tissues (Figures 2(a) and 2(b)). And the expression of linc-ROR in GC tissues was related to the degree of differentiation in clinicopathological factors (Table 1). As a GC tissue-based biomarker, the AUC of linc-ROR reached 0.6495 (Figure 3(a)). The sensitivity and specificity were 0.7524 and 0.5143, respectively (Figure 3(b)). Its false positive rate was 0.4857, and false negative rate was 0.2476. The positive predictive value (PPV) was 0.6077, and negative predictive value (NPV) was 0.675. It was worth noting that the expression of linc-ROR in GC tissues was related to the prognosis of patients with GC (Figure 2(d)). Compared with the low expression linc-ROR group, the high expression linc-ROR group had a better prognosis.

linc-ROR is a lncRNA capable of reprogramming differentiated cells into induced pluripotent stem cells. Studies have shown that linc-ROR can act as a miRNA sponge^40^, ^41^, promote tumor cell proliferation and metastasis, and increase chemotherapy resistance^30^, ^42^, ^43^. For example, Gao et al. found that linc-ROR was significantly upregulated in pancreatic cancer tissues and acted as a ceRNA (competing endogenous RNA) to adsorb miR-145, thereby activating the inhibition of the core transcription factor Nanog^44^. In particular, linc-ROR also can be used as one of the indicators for judging the prognosis of pancreatic cancer^41^. The expression level of linc-ROR was elevated in hepatocellular carcinoma, and the sponge action of linc-ROR on miR-876-5p released FOXM1, thus forming a positive feedback loop. Interestingly, linc-ROR showed the sensitivity to sorafenib in HCC (hepatocellular carcinoma) cell lines^45^. Besides, He et al. examined the expression levels of linc-ROR in liver cancer cell lines and their exosomes, and it was found that linc-ROR was enriched in exosomes of the HepG2 cell line and promoted the growth of other liver cancer cell lines^46^. However, there is no report about the early diagnosis of GC by linc-ROR, so we mainly focus on the diagnostic value of linc-ROR. Our results revealed that linc-ROR has potential as a biomarker for the diagnosis of GC.

In general, linc-ROR is expected to be a tumor diagnostic biomarker for GC, but there are still many shortcomings in this research. Firstly, the quantity of tissue sample is limited, and it is necessary to increase the number of samples in the future research. Secondly, the expression of linc-ROR needs to be detected in blood samples to be more suitable for a tumor biomarker. At present, the combination of biomarkers for early diagnosis of cancer is superior than the single biomarker. Therefore, in the future, we will continue to seek multiple specific markers for GC and build more appreciate models for diagnosis and prognostic evaluation. In addition, the mechanism of linc-ROR in gastric cancer is unclear. Exploring possible molecular mechanisms will help to establish more valuable diagnostic and prognostic biomarkers and provide new molecular targets for the treatment of GC.

## 5. Conclusions

To sum up, our data revealed that linc-ROR expression was significantly decreased in GC tissues. The downregulated linc-ROR expression levels were obviously associated with tumor differentiation, and the high expression linc-ROR group had a better prognosis. Our result suggests that linc-ROR has clinical potential as a biomarker for the diagnosis of GC and assessment of its prognosis.

## Figures and Tables

**Table 1:** The relationship between linc-ROR expression levels (ΔC*q*) in GC tissues and clinicopathological factors of GC patients.

Characteristics	No. of cases (%)	Mean ± SD	*P* value
*Age (years)*			0.324
<60	26 (24.8)	17.6 ± 2.51	
≥60	79 (75.2)	18.09 ± 2.08	
*Gender*			0.748
Male	79 (75.2)	18.01 ± 2.17	
Female	26 (24.8)	17.85 ± 2.29	
*Diameter (cm)*			0.767
<5	45 (42.9)	17.89 ± 1.92	
≥5	60 (57.1)	18.02 ± 2.38	
*Differentiation*			0.004
Well	16 (15.2)	19.51 ± 2.39	
Moderate	53 (50.5)	17.92 ± 2.22	
Poor	36 (34.3)	17.35 ± 1.73	
*Lymphatic metastasis*			0.807
N0	36 (34.28)	18.22 ± 2.03	
N1	19 (18.10)	18.04 ± 1.97	
N2	14 (13.33)	17.82 ± 2.3	
N3	36 (34.29)	17.73 ± 2.15	
*Invasion*			0.675
T1 & TIS	19 (18.10)	17.77 ± 2.18	
T2	11 (10.47)	18.72 ± 1.33	
T3	8 (7.62)	18.06 ± 3.41	
T4	67 (63.81)	17.89 ± 2.15	
*Distal metastasis*			0.811
M0	96 (91.4)	17.98 ± 2.2	
M1	9 (8.6)	17.8 ± 2.2	
*TNM stage*			0.291
I & II	43 (40.9)	18.24 ± 1.97	
III & IV	62 (59.1)	17.78 ± 2.32	
*CEA*			0.392
Positive	92 (87.6)	17.9 ± 2.18	
Negative	13 (12.4)	18.46 ± 2.25	
*CA19-9*			0.509
Positive	61 (58.1)	17.85 ± 2.35	
Negative	44 (41.9)	18.13 ± 1.95	

SD: standard deviation.

**Table 2:** Bioinformatics predicts RBP ( ADAR and FUS ) that can potentially bind to linc-ROR via starBase V3.0.

RBP	Gene ID	Gene name	Gene type
ADAR	ENSG00000258609	linc-ROR	lincRNA
FUS	ENSG00000258609	linc-ROR	lincRNA

RBP: RNA binding protein.

## Data Availability

(1) The quantitative reverse transcription-polymerase chain reaction (qRT-PCR) of linc-ROR data used to support the findings of this study are included within the article. (2) Data and materials availability: all data associated with this study are present in the paper.
